# Cost-Effectiveness of Treatments for Relapsing Remitting Multiple Sclerosis: A French Societal Perspective

**DOI:** 10.1371/journal.pone.0150703

**Published:** 2016-03-17

**Authors:** Julie Chevalier, Catherine Chamoux, Florence Hammès, Annie Chicoye

**Affiliations:** 1 Real World Strategy and Analytics, Mapi Group, Paris, France; 2 Economie de Santé & Affaires Gouvernementales, Biogen France SAS, Nanterre, France; 3 Real World Strategy and Analytics, Mapi Group, Paris, France; University of Oxford, UNITED KINGDOM

## Abstract

**Objectives:**

The paper aimed to estimate the incremental cost-effectiveness ratio (ICER) at the public published price for delayed-release dimethyl fumarate versus relevant Multiple Sclerosis disease-modifying therapies available in France in June 2015.

**Methods:**

The economic model was adapted to the French setting in accordance with the Haute Autorité de Santé guidelines using a model previously developed for NICE. A cohort of Relapsing Remitting Multiple Sclerosis patients was simulated over a 30-year time horizon. Twenty one health states were taken into account: Kurtzke Expanded Disability Status Scale (EDSS) 0–9 for Relapsing Remitting Multiple Sclerosis patients, EDSS 0–9 for Secondary Progressive Multiple Sclerosis patients, and death. Estimates of relative treatment efficacy were determined using a mixed-treatment comparison. Probabilities of events were derived from the dimethyl fumarate pivotal clinical trials and the London Ontario Dataset. Costs and utilities were extracted from the published literature from both the payer and societal perspectives. Univariate and probabilistic sensitivity analyses were performed to assess the robustness of the model results.

**Results:**

From both perspectives, dimethyl fumarate and interferon beta-1a (IFN beta-1a) 44mcg were the two optimal treatments, as the other treatments (IFN beta-1a 30mcg, IFN beta-1b 250mcg, teriflunomide, glatiramer acetate, fingolimod) were dominated on the efficiency frontier. From the societal perspective, dimethyl fumarate versus IFN beta-1a 44mcg incurred an incremental cost of €3,684 and an incremental quality-adjusted life year (QALY) of 0.281, corresponding to an ICER of €13,110/QALY.

**Conclusions:**

Despite no reference threshold for France, dimethyl fumarate can be considered as a cost-effective option as it is on the efficiency frontier.

## Introduction

Multiple sclerosis (MS) is a chronic, progressive, lifelong disease. France suffers one of the highest prevalence of MS in the world, estimated at 1 per 1,000 inhabitants [1]. The mean age of diagnosis is 30 years and 70% of patients are women [1]. MS results in the accumulation of irreversible disability and is the number one disabling disease of young adults. Eighty-five percent of all MS patients suffer from relapsing remitting MS (RRMS) which is characterized by episodes of neurological deterioration (relapses), separated by periods of complete or partial remission [1]. Patients with highly active disease within RRMS is a sub-population that can be characterized as having frequent relapses and progressing more rapidly to severe states of disability and impairment than the broader RRMS population. Patients with MS experience a vast spectrum of symptoms (eg. dysfunctions of vision, motor and sensory systems, coordination and balance, bowel/bladder/sexual and cognition) that vary among relapses or episodes of the disease throughout the progression [2].

Disease severity in MS is quantified using the Kurtzke Expanded Disability Status Scale (EDSS) which measures disability for eight different functional neurological systems (pyramidal, cerebellar, brainstem, sensory, bowel and bladder, visual, cerebral, other) [3]. The extent of disability is quantified according to 20 categories weighted on a scale of 0–10, with 0 being normal neurological examination, and 10 being death due to MS.

Because there is no cure for MS, the goal of treatment is to decrease the frequency and severity of relapses, slow the progression of physical disability, and maintain or improve patients’ health-related Quality of Life (QoL). Most available disease modifying therapies (DMTs) are currently reimbursed for the treatment of RRMS in France. Historically, DMTs were injectable treatments which were burdensome for many patients and had the potential to negatively impact compliance. Injection site reactions, injection fatigue and injection anxiety were shown to impact adherence to treatment, leading to discontinuation [4]. More recently oral treatments have been introduced in France: teriflunomide (2014) in the 1^st^ line setting, fingolimod (2011) in the 2^nd^ line setting and delayed-release dimethyl fumarate (DMF, 2015) in both treatment settings. With a novel mechanism of action, DMF is an innovative approach to RRMS treatment providing patients with a convenient, oral therapy with a favorable risk/benefit profile. DMF has been shown in phase III clinical trials to reduce the frequency of MS relapses by 44–53% and the risk of 3 months sustained disability progression by 21–38% [5, 6, 7, 8]. Indirect comparisons show DMF to be more effective than current first line injectable treatments and teriflunomide, as effective as fingolimod and less effective than natalizumab on relapse rate [9]. The safety profile of DMF has been shown to be favorable, with no difference in the number of serious adverse events compared with placebo [10, 11]. Patients eligible for DMF include naïve patients and those that have failed previous treatment.

An economic model has been developed to estimate the cost-effectiveness of DMF, compared with the competing alternative treatments for RRMS. Given the availability of various treatments with differing efficacy, routes of administration, and costs, the relative cost-effectiveness of these is highly relevant when considering the allocation of resources. Since October 2013 requests for inclusion/renewal of pharmaceuticals in the reimbursement lists in France require a medico-economic assessment when claiming a more than minor additional benefit (ASMR I to III) and a significant impact on the health insurance expenditure or the healthcare organization. The cost-effectiveness analysis described within this paper was submitted to the Commission Evaluation Economique et de Santé Publique (CEESP) at the requested price for DMF and its appraisal is to be published on the website of the French HTA body (Haute Autorité de Santé, HAS).

The aim of this paper is to provide an updated incremental cost-effectiveness ratio (ICER) at the public published price for DMF, following the completion of the procedure for admission to reimbursement.

## Materials and Methods

### Patient population

Patients characteristics at baseline were pooled from the DMF and placebo arms of the pivotal clinical trials DEFINE and CONFIRM [5, 7]. The mean age at entry into the cohort was 37.8 years old and there were 2.5 times more women than men. At baseline, 5.0% of patients were EDSS 0 and 8.5%, 34.1%, 22.9%, 20.6%, 8.6% and 0.1% were EDSS 1, 2, 3, 4, 5 and 6, respectively.

### Model structure

**Fig 1:**
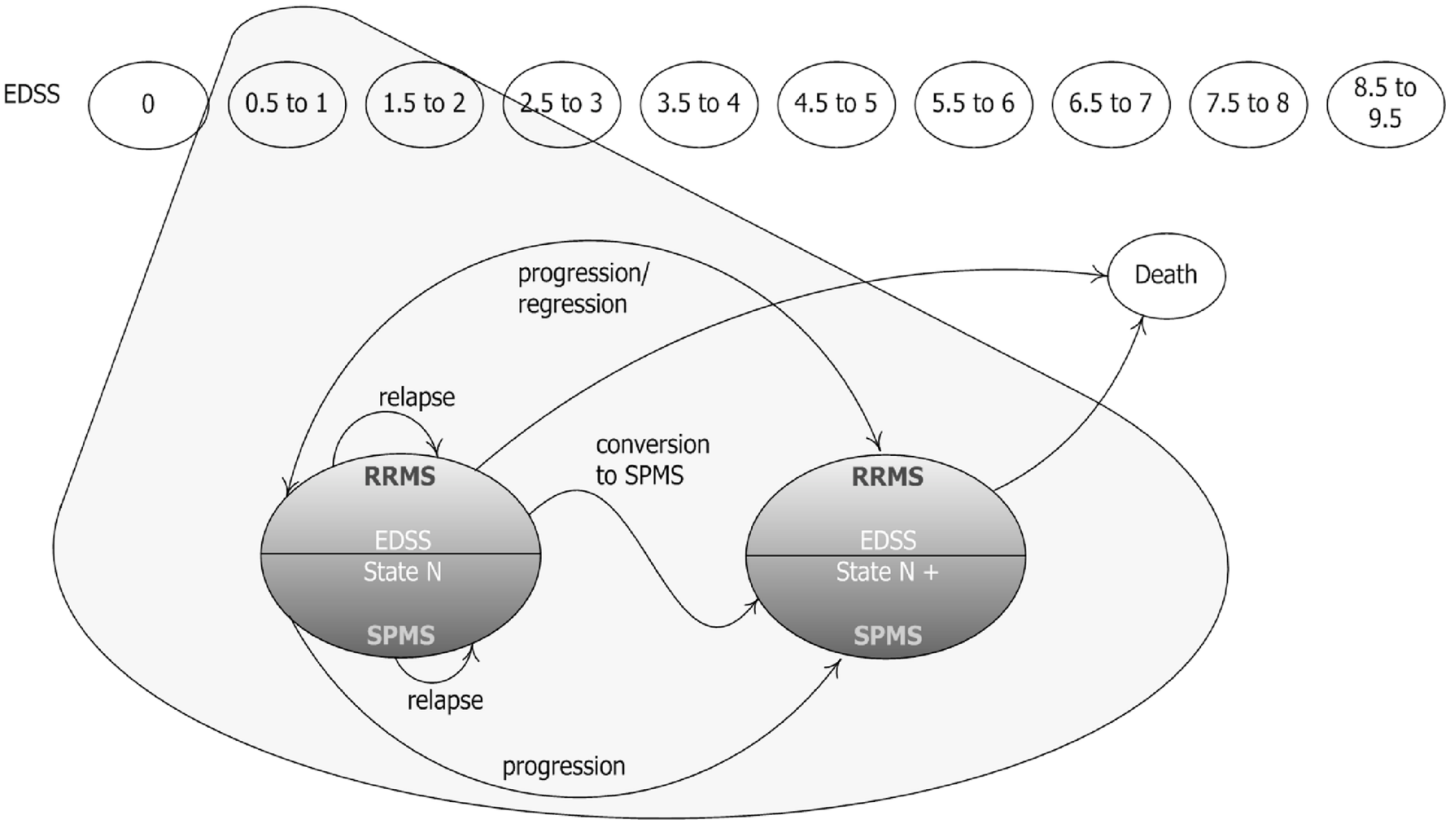
Model schematic.

The economic model employed in this study was adapted to the French setting in accordance with the guidelines of the HAS [12] from a model developed previously for NICE [13]. The cost-effectiveness of DMF was modeled with a 1-year cycle cohort-based Markov model for a theoretical cohort of 1,000 patients. The model considered a time horizon of 30 years as RRMS is a chronic disease and patients would be expected to be on treatment until they progress to Secondary Progressive Multiple Sclerosis (SPMS) or die. The time horizon is in accordance with average life expectancy for MS patients at 39 years of age. Twenty one health states were taken into account (Fig 1): EDSS 0–9 for RRMS patients, EDSS 0–9 for SPMS patients, and death. At each cycle, for patients with RRMS, EDSS scores can increase, decrease or remain the same. In SPMS, EDSS scores can increase or remain the same.

Health and cost outcomes where discounted at 4% per annum during the first 30 years and 2% after as requested by the French guidelines [12].

### Comparator(s)

According to the definition of the patients eligible for DMF, the treatments considered in the model were both first line and second line therapies. DMTs currently reimbursed for the treatment of RRMS in France, in June 2015, are glatiramer acetate, IFNbeta-1a 30mcg intramuscularly and 44mcg subcutaneously, IFN beta-1b 250mcg and teriflunomide as first line therapies and fingolimod and natalizumab, as second line therapies. IFN beta-1a 22mcg was not considered as comparator as it is indicated only as an alternative to the regular dosage of 44mcg in case of intolerance [14]. To take into account all these comparators simultaneously indirect comparisons between placebo and all the different treatments were performed [15]. The analysis was performed excluding natalizumab because it has been shown to be used in more severe patients in France [16].

### Data sources

#### Probability of events

Natural history (without treatment): Disease progression transition probabilities.

To fully evaluate progression of disease, three independent transition probability matrices were used in the model:

- Transition matrix of movement between EDSS states within RRMS (EDSS 0–9)
- Transition matrix of RRMS to SPMS (EDSS 1–9)
- Transition matrix of movement between EDSS states within SPMS (EDSS 1–9)

These matrices are based on the DMF clinical trials placebo arms for EDSS states up to (and including) 7 and the London Ontario dataset [17, 18, 19] for EDSS states 8 and 9 because of the limited number of observations beyond EDSS 8 in the DMF clinical trials. The probability of converting from RRMS to SPMS in each cycle was dependent on current EDSS. These probabilities, as well as EDSS transition matrix for patients with SPMS, were based on data from the London Ontario dataset.

Natural history: annualized relapse rates (ARR): ARR per person per year were sourced from pooled baseline data from the DMF trials, which documented the annual relapse rate in the 12 months before enrolment in the studies.

Mortality rates: Age- and sex-specific all-cause mortality rates for the general population were sourced from the French mortality tables [20]. These mortality rates were then adjusted using the relative risk of death in an MS population, as compared to the general population [21].

**Fig 2:**
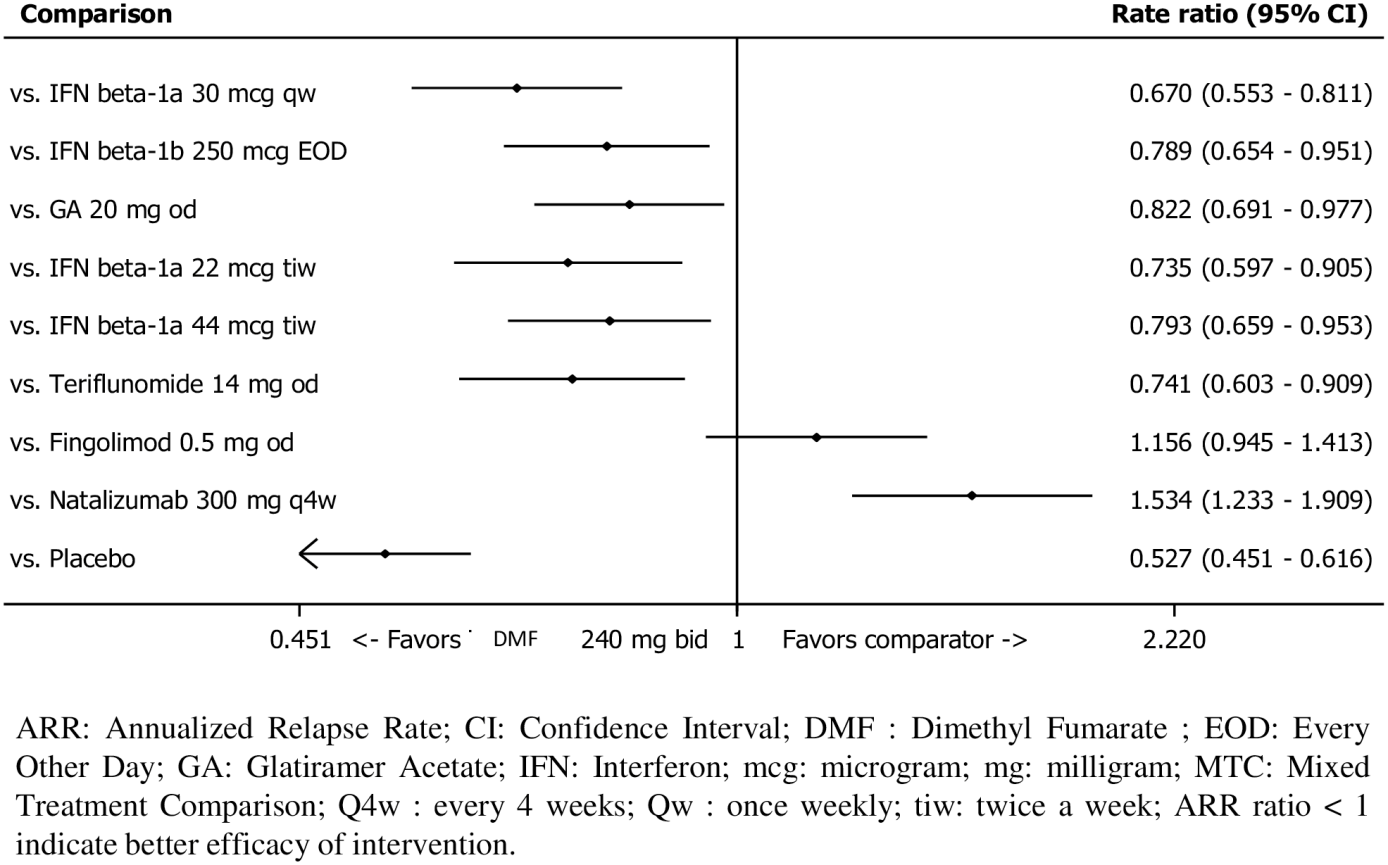
Summary plot of the rate ratio and 95% CIs for MTC of ARR for DMF versus comparators [15].

Efficacy: Estimates of relative treatment efficacy were determined using a mixed-treatment comparison including all available and reimbursed DMTs in France (Fig 2) in June 2015 [9]. The results for the ARR are shown on Fig 2.

Adverse events (AEs): Only the DMF related AEs (identified in the DMF clinical trials) are taken into account in the model. The treatment-specific annual incidence of AEs was calculated from the independent systematic review distinguishing serious and non-serious AEs. The incidence of AEs was assumed to remain constant for all years up to the specified time horizon of the model at a constant rate.

#### Utilities

Utility weights for EDSS states were derived from a publication presenting the EuroQol 5-Dimensions (EQ-5D) utility values of 1,355 French patients [22]. For the purpose of the model, utility results were adapted using the French value set [23] and analyzed for each EDSS stage. No distinction was made between RRMS and SPMS and for the effect of gender. Disutility weights associated with relapse have been calculated and were subtracted from the utility scores for each EDSS [22].

The model evaluates the impact of treatment-related AEs on the accumulation of QALYs by combining the frequency of selected AEs (as observed in the DMF clinical trials), with the assumed disutility. When no reference was available from literature, French experts were consulted to calculate quality of life impact over one annual cycle, the disutility per event was multiplied by the event duration (in years) assessed by experts.

#### Resource use and costs

The baseline scenario considered both direct and indirect costs adopting a societal perspective as accepted by the HAS. This option is justified considering the burden of MS affecting individuals in their thirties and impairing both their personal, social and professional life. A secondary scenario was considered adopting the French Statutory Health insurance payer perspective. The model takes into account costs associated with MS and costs associated with treatment of MS. All costs have been updated to the year 2013 according to the latest available price index of medical services (direct costs) [24] and to the INSEE salaries index (indirect costs) [25] when the model was run. Prices of DMTs have been updated to June 2015.

Treatment-related costs: Treatment-related costs included drug acquisition, administration and monitoring costs, as well as costs related to treatment-related AEs. The cost of medication was based on the French register of pharmaceutical specialities Medicprix [26]. The annual DMTs acquisition costs were calculated for each treatment using the cost per dose multiplied by the number of expected doses per year. Administration, monitoring and AE costs covered medical time, hospitalization, laboratory tests and imaging. Resources used were evaluated by a panel of French experts. Unit costs were sourced from the National Health Tariff [27, 28, 29, 30, 31]. These costs are presented in Table 1. The emergency hospital consultations for AE and Diagnosis Related Group (DRG) codes were sourced from the national (private and public hospital) cost study 2011 (Echelle Nationale de Coûts, ENC) [32].

**Table 1:** Costs (direct and indirect) associated with the treatment of MS (costs presented here are the same in both the payer’s and the societal perspectives).

	DMF	Glatiramer acetate	IFN beta-1a 30mcg	IFN beta-1a 44mcg	IFN beta-1b	Fingolimod	Teriflunomide
Treatment annual acquisition costs	€13,266	€10,809	€10,699	€10,898	€9,244	€24,842	€10,781
Administration costs*	€0.00	€23.70	€23.70	€23.70	€23.70	€0.00	€0.00
1^st^ year	€31.60	€47.40	€47.40	€47.40	€47.40	€361.00	€31.60
Monitoring costs*	€107.54	€53.58	€73.56	€73.56	€73.56	€112.14	€93.54
1^st^ year	€325.64	€251.70	€311.64	€311.64	€311.64	€239.66	€167.10

* For administration and monitoring, costs per year are reported. Due to different administration and monitoring costs during the first year of treatment compared to the following years, these costs are also presented in parenthesis.

EDSS state costs and costs associated with relapses: Resources used per EDSS state and relapses were derived from Kobelt et al. [22]. The direct costs included inpatient care, ambulatory care, tests, prescription drugs other than DMTs, investments in additional resources for care e.g. a wheel chair and services. The indirect costs included short term absence from work, long term sick leave and invalidity pension associated with early retirement. Societal costs include in addition all the other costs, out of pocket expenses or expenses covered by complementary insurance for the 15% of patients non covered by the 100% reimbursement regimen of the statutory health insurance scheme for chronic severe disease (Affection de Longue Durée, ALD) [22]. The revenue losses were valued using the human capital approach and the cost of labor was assumed to represent productivity and was calculated for patients below the official retirement age. Since no statistically significant difference in costs for patients with RRMS, SPMS or PPMS, age and gender was found, no differences in the cost for the different disease stages were applied in the model.

#### Analyses

The base-case model compared DMF to all DMTs (except natalizumab). Model outcomes were QALYs, total costs and ICERs. Treatments were ranked from the cheapest to the most expensive. A treatment less effective than the previous less costly treatment was considered to be strictly dominated and was excluded from the analysis. For each treatment, ICERs were calculated compared with the next non-dominated treatment. A treatment with an ICER higher than that of the next most effective treatment was considered as extendedly dominated and excluded. Treatments not strictly or extendedly dominated made up the efficiency frontier which was drawn by plotting each treatment’s costs and QALYs in a cost-effectiveness plane and connecting the treatment which were not dominated by any of the other treatment.

One-way sensitivity analyses were computed to identify main drivers of cost-effectiveness by varying different parameters individually including: EDSS state costs, relapse costs, patient utilities, natural history relapse rates, treatment relapse rate, treatment disability progression rate, treatment dropouts and treatment costs. Input parameters were arbitrary varied by ± 20% except for EDSS state costs which varied by ± 50% due to the high standard deviation of this parameter. The efficiency frontier was calculated in each sensitivity analysis. A probabilistic sensitivity analysis (PSA) was conducted to compute a cost-effectiveness acceptability curve. The cumulative effect of varying all model parameters within their statistical distributions, based on 95% confidence intervals, was tested. One thousand iterations were run, where each input was sampled at random from probability distribution functions assigned to each variable. We assumed that probabilities had beta distributions, disutilities and relative risks had log-normal distributions and event costs had gamma distributions [33]. The model was computed in Microsoft Excel 2010.

## Results

### Base-case analysis

**Fig 3:**
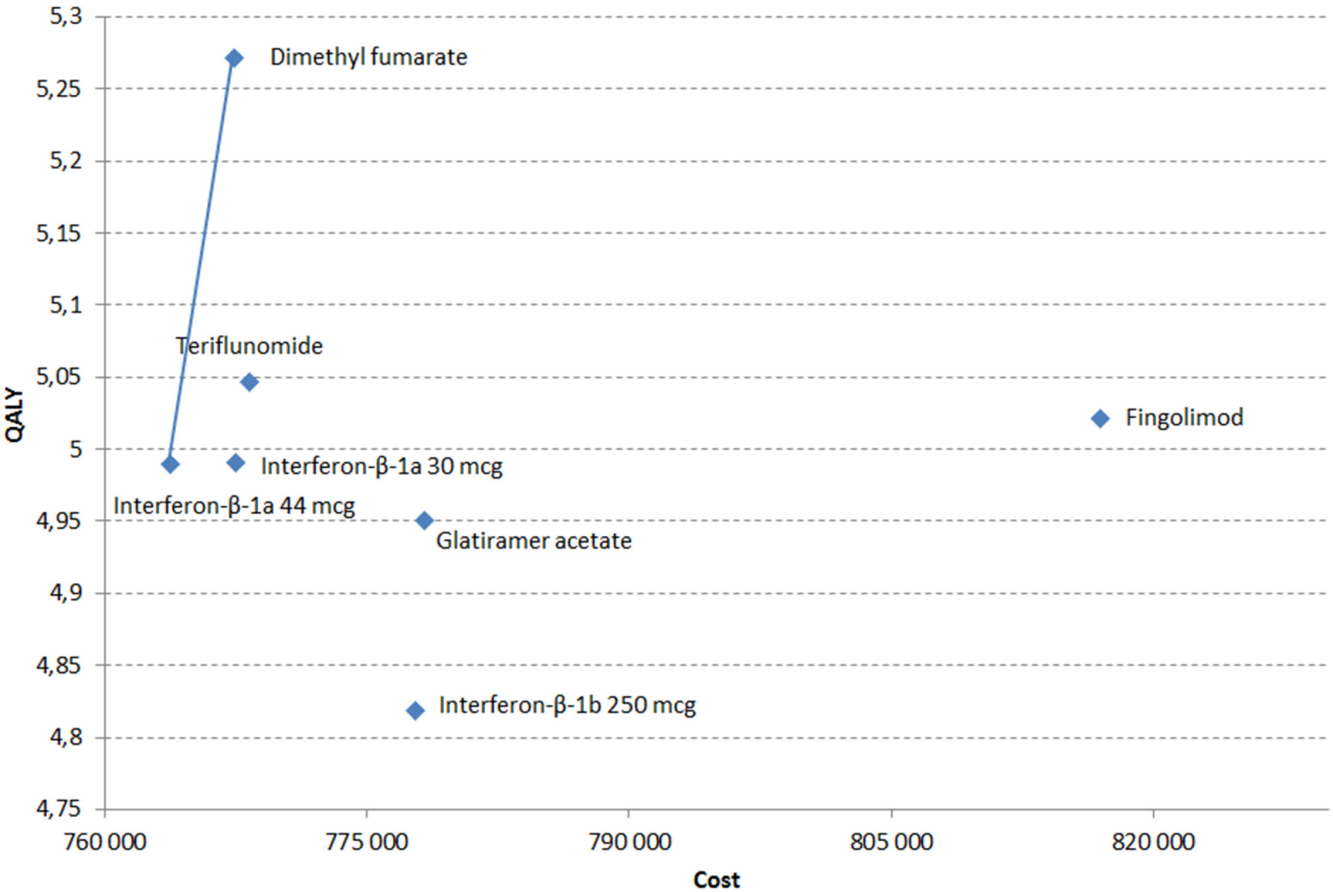
Efficiency frontier comparing treatments.

When considering a societal perspective, total discounted costs amongst the different treatment ranged between €763,790–€816,934 with fingolimod being the treatment associated with the highest total costs mainly because of highest drug costs. The main cost drivers were EDSS state (direct and indirect) costs. Discounted QALYs varied between 4.819–5.271 with DMF associated with the highest number of QALYs. Glatiramer acetate, IFN beta-1a 30mcg, IFN beta-1b 250mcg, fingolimod and teriflunomide were dominated (i.e., higher costs and lower QALYs) by IFN beta-1a 44mcg and DMF (Fig 3). Then IFN beta-1a 44mcg and DMF were on the efficiency frontier, the resulting ICER of DMF vs. IFN beta-1a 44mcg was €13,110/QALY. These results are presented in Table 2.

**Table 2:** Base case results (costs, in € per patient; QALYs): societal perspective.

	DMF	IFN beta-1a 44mcg	IFN beta-1a 30mcg	IFN beta-1b 250mcg	Glatiramer acetate	Teriflunomide	Fingolimod
Costs	€767,474	€763,790	€767,480	€777,775	€778,311	€768,323	€816,934
Treatment costs	€43,540	€27,714	€30,255	€34,662	€36,476	€31,388	€79,419
Administration costs	€648	€561	€544	€676	€506	€422	€837
EDSS state direct costs	€380,342	€388,353	€389,082	€383,182	€392,406	€389,028	€389,377
EDSS state indirect costs	€342,944	€347,162	€347,599	€359,255	€348,922	€347,485	€347,301
QALY	5.271	4.990	4.991	4.819	4.950	5.047	5.021
Incremental results*							
QALY	Reference	- 0.281	- 0.280	- 0.452	-0.321	- 0.224	- 0.250
Costs	Reference	€- 3,684	€6	€10,301	€10,837	€849	€49,460
ICERs	13,110		Dominated	Dominated	Dominated	Dominated	Dominated

* Incremental costs and QALYs are displayed versus DMF. ICERs displayed were calculated using efficiency frontier approach.

When considering a payer perspective, as in the societal perspective, only IFN beta-1a-44mcg and DMF were on the efficiency frontier, the resulting ICER of DMF vs. IFN beta-1a 44mcg was €29,047/QALY.

### One-way sensitivity analyses

**Fig 4:**
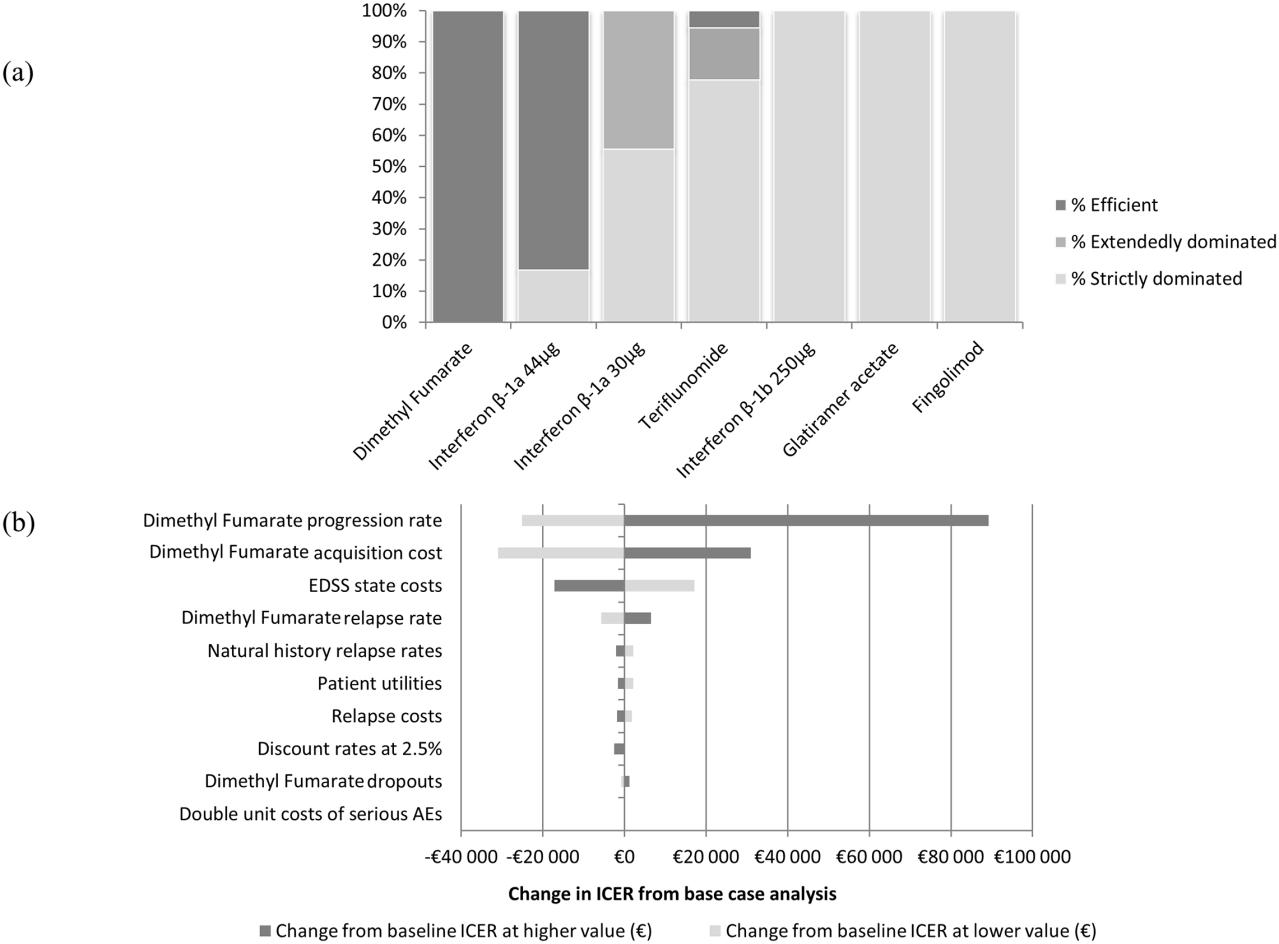
Results of the univariate sensitivity analysis. (a) percentage of scenarios in which each treatment was efficient, extendedly dominated or strictly dominated. (b) Tornado diagram DMF vs. IFN beta-1a 44mcg

In most scenarios (15 out of 18), in the societal perspective, the efficiency frontier was unchanged, with IFN beta-1a 44mcg and DMF being the most efficient treatments and dominating the others (Fig 4). DMF dominated all other treatments including IFN beta-1a 44mcg in 3 scenarios (EDSS state costs +50%, DMF disability progression rate -20% and Price -20%). Teriflunomide appeared in the frontier in only one scenario tested (DMF disability progression rate +20%). The ICER of DMF versus the less costly non-dominated treatment, IFN beta-1a 44mcg in all scenarios but one, ranged between €7,411-€125,164/QALY. Excluding the scenario “DMF disability progression rate +20%”, DMF was always compared to IFN beta-1a 44mcg with a maximum ICER of €44,011/QALY.

The one way sensitivity analysis of DMF versus IFN beta-1a 44mcg, i.e. the only non-dominated alternative showed the ICER of DMF versus IFN beta-1a 44mcg varies between €-30,927-€89,252/QALY comparatively to the base-case value; ie. between €-16,842–€76,167/QALY. The ICER was most influenced by DMF disability progression rate, DMF acquisition cost, EDSS state cost and DMF relapse rate (Fig 4).

Results of the one-way sensitivity analysis are similar in the payer perspective.

### Probabilistic sensitivity analysis

**Fig 5:**
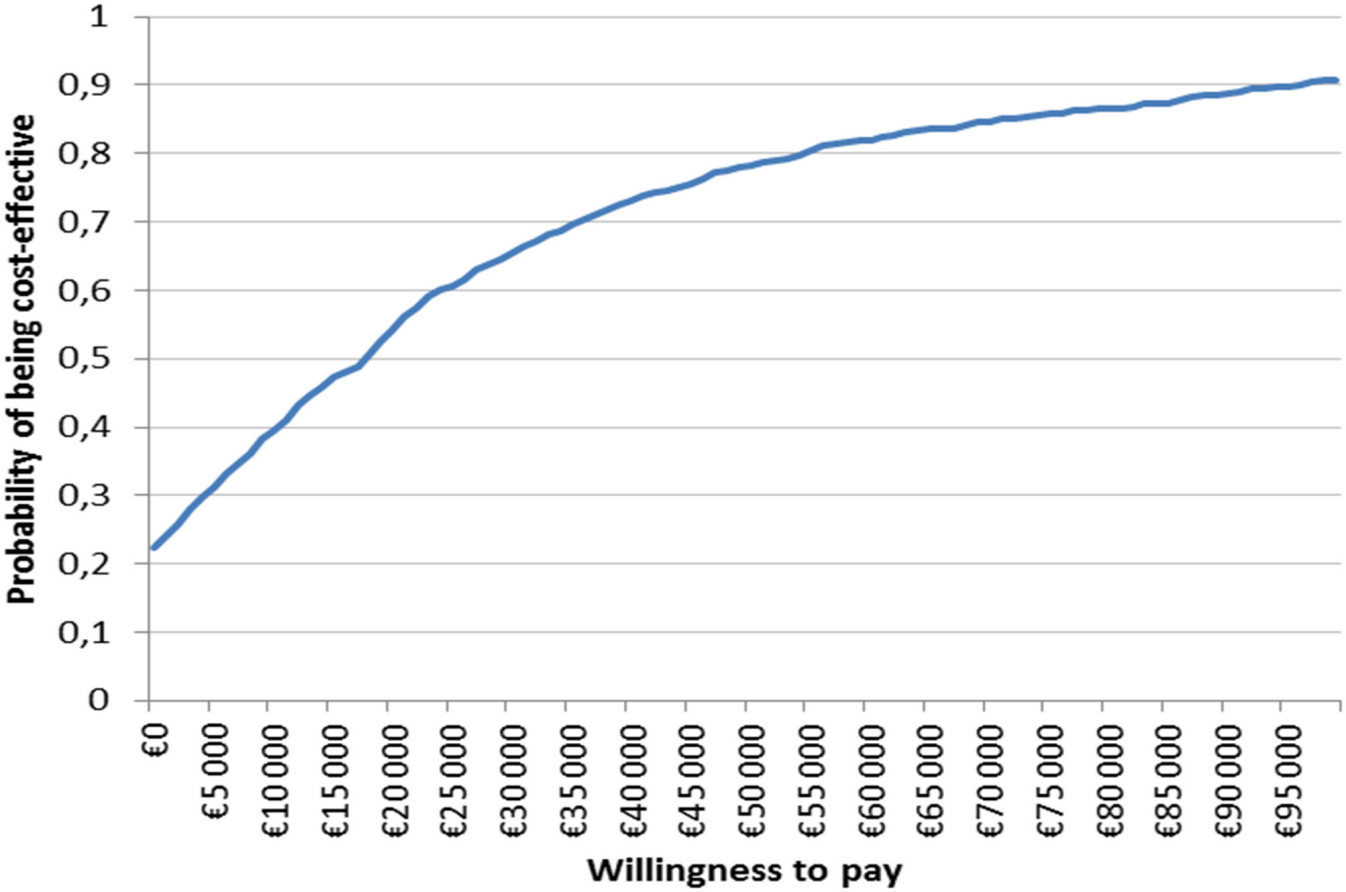
Cost-effectiveness acceptability curve of DMF versus IFN beta-1a 44mcg (non-dominated strategy).

The results of the PSA showed a great variability in the results. The ICER of DMF was <€30,000 in 65% of the simulations and <€100,000 in 91% (Fig 5) in the societal perspective. Considering the payer perspective, the ICER of DMF was <€30,000 in 46% of the simulations and <€100,000 in 91%.

## Discussion

This economic evaluation is the first to compare DMF to all its currently available comparators in the treatment of MS in France. As no cost-effectiveness threshold is determined, the HAS recommends in its guidelines [12] to present the different treatments in term of dominance through the efficiency frontier. In the base-case analysis (societal perspective), only cost-effective treatments were compared to each other (DMF vs IFN beta-1a 44mcg). The ICER of DMF compared to IFN beta-1a 44mcg was €13,110/QALY (€29,047/QALY in the payer perspective). The base-case analysis demonstrated that DMF dominated all other treatments except IFN beta-1a 44 mcg. Univariate sensitivity analysis demonstrated that DMF was consistently the most cost-effective treatment. The results of the PSA (DMF compared to IFN beta-1a 44mcg) showed a great variability in the results.

Using the same model structure, a similar cost-effectiveness analysis was submitted to the CEESP which is in charge of assessing innovative health technologies in France with the requested price for DMF. The CEESP appraised favorably the methodology of the analysis but highlighted the lack of consideration of teriflunomide and alemtuzumab in the model. To date, alemtuzumab is not marketed. The present analysis included teriflunomide, meeting one of the CEESP critics.

As emphasized by the CEESP, the model does not aim at simulating the successive sequences of treatment, as the objective is to assess the relative ICER of DMF versus alternatives relevant for various types of RRMS and depending upon the treatment history. However, another model will be developed to take the sequences of treatment into account.

Other limitations could be noted. First, the AEs included in the model are only those of DMF. This is a conservative assumption, and is likely to bias the results of the analysis against DMF. In addition, serious AEs were not considered in the model for IFN beta-1a 44mcg and IFN beta-1b, this option being also conservative for DMF. It is assumed that a patient who receives treatment will incur the risk of disutility and cost associated with AEs, for each year in the simulation. This may overestimate the impact of AEs attributable to DMF versus its comparators, as AEs of DMF are transient and experienced mainly in the first 24 months. Second, all costs, except for treatment, have been calculated in €2013 which is the last year where price index of medical services and INSEE salaries index are available. On the other hand we updated the prices of DMTs to June 2015 as a price-cut occurred for IFN beta-1a 44mcg and 30mcg in May. According to the deterministic sensitivity analysis, the EDSS state costs had an impact on the ICER but this impact does not change the rough size of the ratio and there is no reason to say that the costs taken into account in the model should be significantly changed between 2013 and 2015.

## Conclusion

To conclude, DMF can be considered as a cost-effective option compared to other DMTs.
